# Efficacy and safety profile of doxofylline compared to theophylline in asthma: a meta-analysis

**DOI:** 10.1186/s40248-019-0189-0

**Published:** 2019-08-03

**Authors:** Paola Rogliani, Luigino Calzetta, Josuel Ora, Mario Cazzola, Maria Gabriella Matera

**Affiliations:** 1 Unit of Respiratory Medicine, Department of Experimental Medicine, University of Rome “Tor Vergata”, Via Montpellier 1, 00133 Rome, Italy; 2 Division of Respiratory Medicine, University Hospital “Tor Vergata”, Rome, Italy; 3 Unit of Pharmacology, Department of Experimental Medicine, University of Campania “Luigi Vanvitelli”, Naples, Italy

**Keywords:** Asthma, Doxofylline, Theophylline, Meta-analysis

## Abstract

**Background:**

Oral methylxanthines are effective drugs for the treatment of chronic obstructive respiratory disorders. The novel methylxanthine doxofylline, that has bronchodilator and anti-inflammatory activities, is not affected by the major drawback of theophylline. Nowadays large-scale quantitative synthesis comparing the efficacy and safety profile of doxofylline vs. theophylline in the treatment of asthma is still lacking. Therefore, we performed a quantitative synthesis to compare the efficacy/safety profile of doxofylline and theophylline in asthma.

**Methods:**

A pairwise and network meta-analyses were performed to assess the impact of doxofylline vs. theophylline and placebo on the change in asthma events, risk of adverse events (AEs), forced expiratory volume in 1 s (FEV_1_), and salbutamol use.

**Results:**

Data obtained from 696 asthmatic patients were extracted from 4 randomized controlled trials published between 2015 and 2018. Doxofylline was significantly (*P* < 0.05) more effective than theophylline in reducing the daily asthma events (mean difference − 0.14, 95%CI -0.27 – 0.00) and risk of AEs (relative risk 0.76, 95%CI 0.59–0.99). Doxofylline was as effective as theophylline in improving FEV_1_, and a trend of superiority (*P* = 0.058) was detected for doxofylline over theophylline with respect to the reduction in the use of salbutamol as rescue medication. The rank of effectiveness was doxofylline>theophylline> > placebo, and the rank of safety was placebo>doxofylline> > theophylline.

**Conclusions:**

Doxofylline is an effective and safe methylxanthine for the treatment of asthma, with an efficacy/safety profile greater than that of theophylline.

**Trial registration:**

Meta-analysis registration: CRD42019119849.

## Background

Oral methylxanthines are recognized effective drugs for the clinical management of patients suffering from chronic obstructive respiratory disorders. The novel methylxanthine doxofylline, that is characterized by bronchodilator and anti-inflammatory activities, seems to be not affected by the major drawback of theophylline [1–5]. Doxofylline has a favourable efficacy profile accompanied by a high level of tolerability, at least in COPD patients [6]. Moreover, an observational study aimed to assess the treatment plan in acute and chronic respiratory tract diseases, has demonstrated a significant difference in the unit cost per patient in favour of doxophylline compared to theophyllne. In fact, although the cost of doxophylline was higher than that of theophyllne, doxofylline resulted to be associated with a reduction of the overall cost related with COPD and/or asthma management [7].

Recently, the pooled analysis of two multicenter, double-blind, randomized trials, carried out in 38 US pulmonary clinics that investigated the therapeutic efficacy and tolerability of doxofylline compared to theophylline, demonstrated that doxofylline is an effective and well tolerated agent in asthmatic patients [8]. Although the beneficial efficacy/safety profile of doxofylline has been reported in further smaller clinical trials and retrospective studies [9–14], to date large-scale quantitative synthesis comparing the efficacy and safety profile of doxofylline vs. theophylline in the treatment of asthma is still lacking.

The hypothesis of this study is that a quantitative synthesis of clinical trials that directly compared the efficacy and safety of doxofylline vs. theophylline may provide more robust evidences than individual studies or pooled analyses. Therefore, we performed a pairwise and network meta-analysis to definitively clarify which of the two drugs should be prescribed when a methylxanthine is recommended in asthmatic patients.

## Methods

### Search strategy

**Fig. 1:**
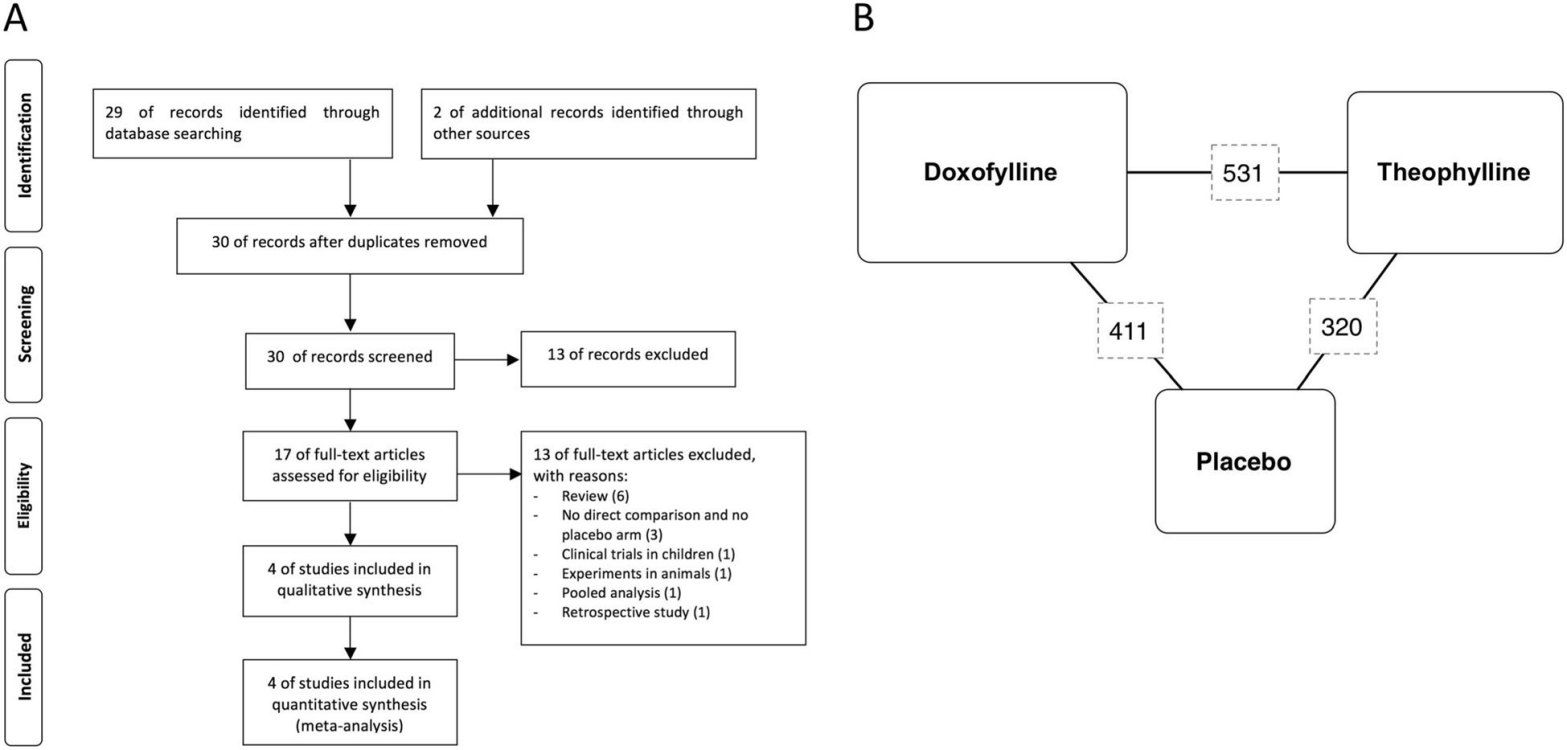
PRISMA flow diagram for the identification of studies included in the meta-analysis (**a**) and diagram displaying the network across the treatments; the links between nodes indicate the direct comparisons between pairs of treatments; the numbers shown along the link lines indicate the number of patients comparing pairs of treatments head-to-head (**b**)

This meta-analysis has been submitted to the international database of prospectively registered systematic reviews (PROSPERO, registration number: CRD42019119849), and performed in agreement with the Preferred Reporting Items for Systematic Reviews and Meta-Analyses Protocols (PRISMA-P) [15]. The PRISMA flow diagram is reported in Fig. 1a. This quantitative synthesis satisfied all the recommended items reported by the PRISMA-P checklist [15].

Two reviewers performed a comprehensive literature search for randomized controlled trials (RCTs) evaluating the influence of doxofylline and theophylline in adolescent and adult asthmatic patients. The PICO (Patient problem, Intervention, Comparison, and Outcome) framework was used to develop the literature search strategy, as previously described [16]. Namely, the “Patient problem” included subject affected by asthma; the “Intervention” regarded the administration of doxofylline and theophylline; the “Comparison” was performed with regard to placebo and across each active treatment; the “Outcomes” were the asthma events, adverse events (AEs), lung function expressed as forced expiratory volume in 1 s (FEV_1_), and use of salbutamol as rescue medication.

The terms “doxofylline” AND “theophylline” AND “asthma” were searched in Cochrane Central Register of Controlled Trials (CENTRAL), MEDLINE, Embase, Scopus, Web of Science, ClinicalTrials.gov and EU Clinical Trials Register databases in order to provide for relevant studies available up to December 7, 2018. No language restriction was applied. The following Query Translation was used: (“asthma” [MeSH Terms] OR “asthma”[All Fields]) AND (“theophylline” [MeSH Terms] OR “theophylline” [All Fields]) AND (“doxofylline” [Supplementary Concept] OR “doxofylline”[All Fields]).

Citations of previously published analyses and relevant reviews were examined to identify further pertinent studies, if any [6, 8, 17–19].

### Study selection

Published RCTs involving asthmatic patients treated with oral formulations of doxofylline and theophylline were included in this meta-analysis.

Two reviewers independently checked the relevant studies identified from literature searches obtained from the already mentioned databases. The studies were selected in agreement with the above-mentioned criteria, and any difference in opinion about eligibility was resolved by general consensus.

### Endpoints

The primary endpoints of this meta-analysis were the impact of doxofylline on the change in asthma events and the risk of AEs, compared to theophylline and placebo. Asthma events were defined as episodes of increased symptoms or increased airflow limitation responsive to as needed short-acting β_2_ agonists or resulting in either unscheduled medical attention, unscheduled use of oral corticosteroids, hospital admission [20]. The secondary endpoints were the impact of doxofylline on the change in FEV_1_ and salbutamol use, compared to theophylline.

### Quality score, risk of bias and evidence profile

The Jadad score, with a scale of 1 to 5 (score of 5 being the best quality), was used to assess the quality of the RCTs concerning the likelihood of biases related to randomization, double blinding, withdrawals and dropouts [21]. A Jadad score ≥ 3 was defined to identify high quality studies. Two reviewers independently assessed the quality of individual studies, and any difference in opinion about the quality score was resolved by consensus.

In the pairwise meta-analysis moderate to high levels of heterogeneity between-studies were considered for I^2^ > 50%; the risk of publication bias was assessed by applying the funnel plot and Egger’s test, as previously described [21]. Evidence of asymmetry from Egger’s test was considered to be significant at *P* < 0.1, and the graphical representation of 90% confidence bands have been presented [21]. The risk of bias in the network meta-analysis was checked via the consistency/inconsistency analysis to assess whether the outcomes resulting from the consistency and inconsistency models fit adequately with the line of equality, as previously described [22].

The quality of the evidence was assessed for the primary endpoint in agreement with the Grading of Recommendations Assessment, Development, and Evaluation (GRADE) system, indicating ++++ for high quality of evidence, +++ for moderate quality of evidence, ++ for low quality of evidence, and + for very low quality of evidence [23].

### Data extraction

Data from included RCTs were extracted and checked for study characteristics and duration, enrolled patients, treatments and doses, disease characteristics, age, gender, asthma events, previous hospitalizations, lung function, and Jadad score. Data have been extracted in agreement with DECiMAL recommendations [24].

### Data analysis

A pairwise meta-analysis was performed to quantify the impact of doxofylline compared to theophylline on primary and secondary endpoints.

Results of the pairwise meta-analysis are expressed as mean difference (MD) or relative risk (RR), and 95% confidence interval (95%CI). Since data were selected from a series of studies performed by researchers operating independently, and a common effect size cannot be assumed, binary random-effects model was used in order to balance the study weights and adequately estimate the 95%CI of the mean distribution of drugs effect on the investigated variables [21].

A network meta-analysis was also carried out to perform a comparison across doxofylline, theophylline, and placebo with respect to the primary endpoints, and to rank their efficacy in reducing asthma events and the risk of AEs.

The network meta-analysis was carried out by including RCTs that introduced no significant heterogeneity and bias in the effect estimates of primary endpoint. Since heterogeneity and bias may propagate through a network of RCTs, and thus affect the estimates differentially across regions of the network, this approach permitted to identify those studies that might alter the correct results of the network meta-analysis [25].

Full Bayesian evidence network was used in the network meta-analysis (chains: 4; initial values scaling: 2.5; tuning iterations: 20.000; simulation iterations: 50.000; tuning interval: 10). The convergence diagnostics for consistency and inconsistency was assessed via the Brooks-Gelman-Rubin method, as previously described [26]. Results of the network meta-analysis are expressed as relative effect (RE) and 95% credible interval (95%CrI). The probability that each intervention arm was the most effective was calculated by counting the proportion of iterations of the chain in which each intervention arm had the highest mean difference, and the surface under the cumulative ranking curve (SUCRA), representing the summary of these probabilities, was also calculated. The SUCRA is 1 when a treatment is certain to be the best, and 0 when a treatment is certain to be the worst [27].

Sensitivity analysis was performed to identify the studies that introduced heterogeneity and bias in the effect estimate of primary endpoints, if any. Meta-regression analysis was performed to identify the factors that might modulate the efficacy and safety profile of the investigated agents with regard to primary endpoints.

The specisafety profile of the investigated treatments was investigated through a pooled analysis of the frequency of specific AEs.

OpenMetaAnalyst [28] and GeMTC [29] software were used for performing the meta-analysis, GraphPad Prism (CA, US) software to graph the data, and GRADEpro GDT to assess the quality of evidence [23]. The statistical significance for the effect estimates resulting from the pairwise and network meta-analyses was assessed for *P* < 0.05.

## Results

### Studies characteristics

Data obtained from 696 asthmatic patients (44.68% treated with doxofylline, 31.62% treated with theophylline, and 23.71% treated with placebo) were selected from 4 RCTs published between 2015 and 2018 [8–10]. The relevant characteristics of studies, disease, and patients are described in Table 1; Fig. 1b shows the network across the treatments.

**Table 1:** Patient demographics, baseline and study characteristics

Study, year, trial registration, and reference	Study characteristics	Study duration (weeks)	Enrolled patients	Treatments and doses	Concomitant medications for asthma	Disease characteristics	Age (years)	Male (%)	Asthma events (n/day)	Previous hospitalization for asthma (%)	FEV_1_ (% predicted)	Jadad score
Calzetta et al., 2018, DOROTHEO 1, ISRCTN65297911, http://www.isrctn.com/ISRCTN65297911, [8]	Multicentre, double-blind, randomized, placebo-controlled, parallel-group	12	346	Doxofylline 200 mg t.i.d., doxofylline 400 mg t.i.d., theophylline 250 mg t.i.d., placebo	Salbutamol as needed	FEV_1_ ≥ 50 and < 80%, ≥15% post-bronchodilator increase in FEV_1_	36	49	1.2	40	66	5
Calzetta et al., 2018, DOROTHEO 2, ISRCTN65297911, http://www.isrctn.com/ISRCTN22374987, [8]	Multicentre, double-blind, randomized, placebo-controlled, parallel-group	12	220	Doxofylline 400 mg t.i.d., theophylline 250 mg t.i.d., placebo	Salbutamol as needed	FEV_1_ ≥ 50 and < 80%, ≥15% post-bronchodilator increase in FEV_1_	37	45	1.0	42	67	5
Lal et al., 2015, NA, [10]	Single-centre, open, parallel-group	8	30	Doxofylline 200 mg b.i.d., theophylline 200 mg b.i.d.	Standard treatment	NA	35	NA	NA	NA	NA	1
Margay et al., 2015, NA, [9]	Single-centre, open, randomized, parallel-group	6	100	Doxofylline 400 mg b.i.d., theophylline 300 mg b.i.d.	Salbutamol as needed	FEV_1_ ≥ 50% and ≤ 80%, ≥12% post-bronchodilator increase in FEV_1_	38	45	1.7	NA	68	3

*FEV*_1_ forced expiratory volume in 1 s, *NA* not available

All the RCTs were published as full-text papers [8–10]. The DOROTHEO 1, DOROTHEO 2, and the study of Margay et al. were published as high quality studies (Jadad score ≥ 3) [8, 9], whereas the study of Lal et al. as low-quality study (Jadad score = 1) [10]. The duration of treatment ranged from 6 weeks to 12 weeks.

### Primary endpoints

**Fig. 2:**
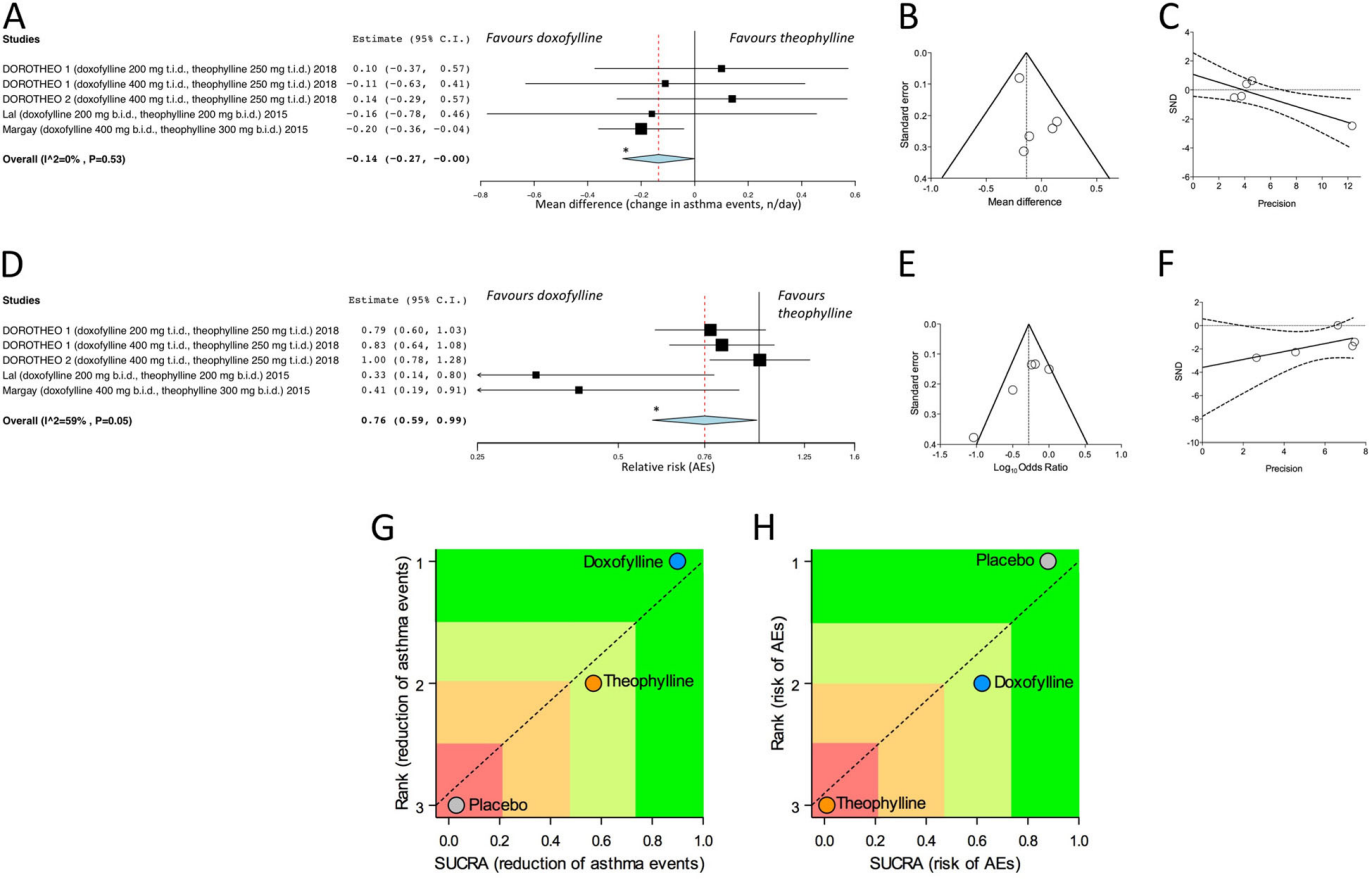
Forest plot of pair-wise meta-analysis concerning the impact of doxofylline vs. theophylline on asthma events (**a**) and adverse events (**d**), with relative publication bias assessment via funnel plots (**b** and **e**) and Egger’s test (**c** and **f**). Ranking plot resulting from the network meta-analysis of efficacy (**g**) and safety (**h**) in which treatments were plotted on X-axis according to SUCRA (score of 1 being the most effective) and on Y-axis according to the rank of being the best treatment (score of 1 being the most effective). *P < 0.05. AEs: adverse events; SND: standard normal deviate; SUCRA: surface under the cumulative ranking curve

The pairwise meta-analysis indicated that doxofylline was significantly (*P* < 0.05) more effective than theophylline in reducing the daily asthma events (MD -0.14, 95%CI -0.27 – 0.00) (Fig. 2a). The effect estimate was not affected by heterogeneity (I^2^ 0%, *P* = 0.53). Although a certain level of asymmetry resulted by the visual inspection of funnel plot, the Egger’s test found not significant publication bias (Fig. 2b and c). The risk of AEs was significantly (*P* < 0.05) lower in patients treated with doxofylline than in those that received theophylline (RR 0.76, 95%CI 0.59–0.99) (Fig. 2d). The substantial but not significant heterogeneity (I^2^ 59%, *P* = 0.05) was confirmed by the visual inspection of funnel plot (Fig. 2e), and mainly related with the study of Lal et al. [10]. However, the analysis performed via Egger’s test indicated that, although the study of Lal et al. [10] was small and characterized by low quality, it introduced no significant publication bias in the effect estimate concerning the risk of AEs (Fig. 2f).

The results of the network meta-analysis indicated that doxofylline, but not theophylline, significantly (*P* < 0.05) reduced the daily asthma events (RE: − 0.33, 95%CrI -0.62 - -0.04, − 0.33; − 0.23 95%CrI -0.54 - 0.04; respectively, compared to placebo). Doxofylline was also safer than theophylline, reporting a significant (P < 0.05) reduction in the risk of AEs (RE 0.53, 95%CrI 0.27–0.87; doxofylline vs. theophylline). The network meta-analysis also showed that doxofyline was the most effective treatment (upper quartile in the SUCRA ranking) with respect to both theophylline (second quartile in the SUCRA ranking) and placebo (lower quartile in the SUCRA ranking) (Fig. 2 g). As expected, placebo was the safest arm (upper quartile in the SUCRA ranking), followed by doxofylline (second quartile in the SUCRA ranking); conversely, theophylline was the less safe treatment (lower quartile in the SUCRA ranking) (Fig. 2 h).

The consistency/inconsistency analysis showed that all points fit adequately with the line of equality (efficacy: R^2^ 0.99; slope 1.05, 95%CI 0.96–1.15), indicating that the network meta-analysis was not affected by significant bias.

Meta-regression analysis confirmed that the patient demographics, baseline and study characteristics did not modulate the efficacy and safety profile of the investigated agents.

The GRADE analysis indicated high quality of evidence (++++) for doxofylline vs. theophylline with respect to efficacy and safety profile in both pairwise and network meta-analysis.

### Secondary endpoints

**Fig. 3:**
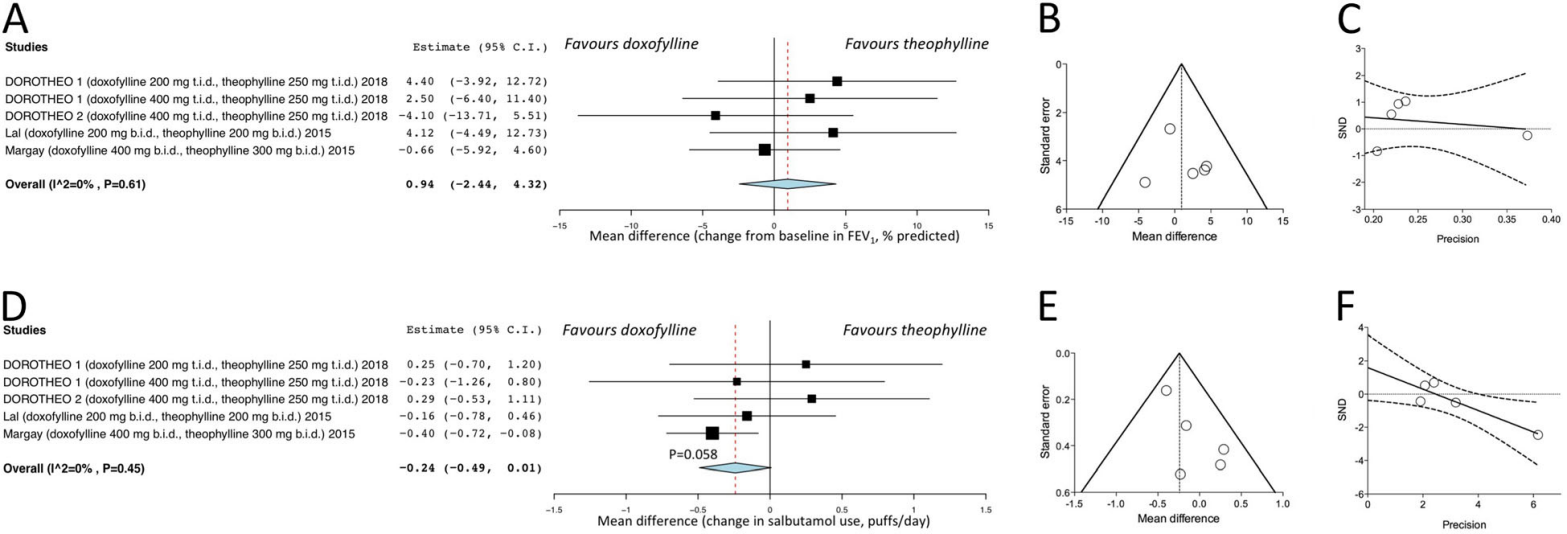
Forest plot of pair-wise meta-analysis concerning the impact of doxofylline vs. theophylline on the change in FEV_1_ (**a**) and salbutamol use as recue medication (**d**), with relative publication bias assessment via funnel plots (**b** and **e**) and Egger’s test (**c** and **f**). FEV_1_: forced expiratory volume in 1 s; SND: standard normal deviate

There was no significant difference between doxofylline and theophylline on the change in change in FEV_1_ (*P* > 0.05) (Fig. 3a). A trend of superiority (*P* = 0.058) was detected for doxofylline over theophylline with respect to the reduction in the use of salbutamol as rescue medication (Fig. 3b).

The pooled analysis of safety profile showed that the AEs with a frequency greater than 5% were headache (doxofylline 20.61%, theophylline 23.64%), nausea (doxofylline 10.96%, theophylline 21.82%), nervousness (doxofylline 4.39%, theophylline 11.36%), and dyspepsia (doxofylline 6.58%, theophylline 8.18%). AEs were generally mild in severity, and detailed frequencies of further specific AEs are reported in Table 2.

**Table 2:** Pooled analysis of AEs (sorted by descending order) extracted from the studies that directly compared doxofyline with theophylline in asthmatic patients

	Doxofylline		Theophylline	
Total number of asthmatic patients	228		220	
	%	n	%	n
Asthmatic patients reporting at least one AE	46.05	105	57.73	127
Total number of AEs				
headache	20.61	47	23.64	52
nausea	10.96	25	21.82	48
nervousness	4.39	10	11.36	25
insomnia	6.14	14	9.55	21
dyspepsia	6.58	15	8.18	18
vomiting	3.51	8	4.09	9
dizziness	3.95	9	3.18	7
cough increased	0.44	1	3.64	8
overdose	0.00	0	3.64	8
rhinitis	3.51	8	2.73	6
diarrhoea	3.07	7	3.18	7
asthma	3.07	7	3.18	7
abdominal pain	2.63	6	1.82	4
pharyngitis	1.32	3	2.27	5
palpitations	2.19	5	1.82	4
chest pain	2.19	5	0.00	0
epigastric distress	0.88	2	1.82	4
asthenia	0.00	0	1.82	4
tremors	0.00	0	0.91	2
somnolence	0.44	1	0.45	1
sore throat	0.00	0	0.45	1

*AE* adverse event

## Discussion

This meta-analysis showed that treatment with doxofylline was significantly more effective than theophylline in reducing the daily asthma events and preventing the risk of AEs, which were the primary endpoints of this meta-analysis. As expected, the SUCRA analysis performed by considering high quality RCTs reported that doxofylline had a better efficacy profile than both theophylline and placebo, and that theophylline was ranked as the less safe treatment in this quantitative synthesis. Interestingly, the pooled analysis indicated that the percentage of the most frequently recorded AEs (i.e. headache, nausea, nervousness, insomnia, dyspepsia, and vomiting) was generally greater in asthmatic patients treated with theophylline than in those that received doxofylline. Overall, the results of this study are strong, as they were not affected by publication bias, and with high quality of evidence for both the pariwise and network meta-analyses.

Concerning the secondary endpoints, doxofylline was as effective as theophylline in improving FEV_1_, although a trend toward significance [30] suggested that doxofylline was superior than theophylline concerning the reduction in the use of salbutamol as rescue medication.

Although significant, the difference in efficacy outcomes between doxofylline and theophylline did not reach the minimum clinically important differences (MCIDs), when considering the comparison across active treatments [31]. Nevertheless, in the network meta-analysis the range of protection against the risk of AEs was clinically meaningful for doxofylline vs. theophylline, as the 95%CrI of RR was prevalently below the 0.75 value, with the mean set at RR 0.53 [32]. The small differences in results between the pairwise and network meta-analyses may be due to the presence of the placebo node in the Bayesian analysis that reinforced the comparison across the investigated arms.

Taken together the findings of this meta-analysis support the rationale for using doxofyilline to treat chronic obstructive respiratory disorders, and its superiority with respect to theophylline [6, 8, 17]. Considering also the beneficial cost-effectiveness profile of the pharmacological treatment with doxofyilline [7], and the advantage of not needing the monitoring of theophyllinemia, there is no reason not to choose doxofylline as first line treatment when a methylxanthine is indicated in asthmatic patients.

Indeed, meta-analyses have evolved as a technique useful for summarizing the evidences form a large number of RCTs and for resolving discrepancies raised by clinical studies. Nevertheless, meta-analyses mainly deal with populations and not with single patients [33]. In this respect, the use of an effective, safe and inexpensive orally active drug as doxofylline should be encouraged especially in those patients who find inhalers difficult to use. The use of doxofylline, instead of theophylline, may have a strong rationale also in those patients who do not get adequate control from other pharmacological classes, such as inhaled corticosteroids in smokers asthmatics or β_2_-adrenoceptor agonists in subjects with a genetic polymorphism resulting in homozygosity for arginine at amino acid residue 16 of the β_2_-adrenergic receptor [34, 35].

Finally, but not less important, the current evidence clearly indicates that the Global Initiative for Asthma (GINA) recommendations [36] should be updated by considering doxofylline as a more effective and safer alternative to theophylline in Step 1 to 4 treatments, and as optional treatment in acute care settings.

## Conclusions

The results of this quantitative synthesis of the current literature proves that doxofylline is an effective and safe methylxanthine for the treatment of asthma, and that its efficacy/safety profile is greater than that of theophylline.

## Data Availability

All data generated or analysed during this study are included in this published article.

## Abbreviations

AEs: adverse events
CENTRAL: Cochrane Central Register of Controlled Trials
CI: confidence interval
CrI: credible interval
FEV_1_: forced expiratory volume in 1 s
GINA: Global Initiative for Asthma
GRADE: Grading of Recommendations Assessment, Development, and Evaluation
MD: mean difference
PICO: Patient problem, Intervention, Comparison, and Outcome
PRISMA-P: Preferred Reporting Items for Systematic Reviews and Meta-Analyses Protocols
PROSPERO: prospectively registered systematic reviews
RCTs: randomized controlled trials
RE: relative effect
RR: relative risk
SUCRA: surface under the cumulative ranking curve
